# Potential Anti-Tumor Activity of Kefir-Induced Juglone and Resveratrol Fractions Against Ehrlich Ascites Carcinoma-Bearing BALB/C Mice

**DOI:** 10.22037/ijpr.2020.112993.14060

**Published:** 2020

**Authors:** Erhan Bozkurt, Emre Atay, Gökhan Pektaş, Ayşe Ertekin, Ayhan Vurmaz, Ömer Adil Korkmaz, Gökhan Sadi, Esra Aslan, Oğuz Han Koca, Mehmet Bilgehan Pektaş

**Affiliations:** a *Department of Internal Medicine, Faculty of Medicine, Afyonkarahisar Health Sciences University, 03200, Afyonkarahisar, Turkey. *; b *Department of Anatomy, Faculty of Medicine, Afyonkarahisar Health Sciences University, 03200, Afyonkarahisar, Turkey. *; c *Department of Hematology, Faculty of Medicine, Mugla Sitki Kocman University, 48000, Muğla, Turkey. *; d *Department of Emergency Medicine, Faculty of Medicine, Afyonkarahisar Health Sciences University, 03200, Afyonkarahisar, Turkey. *; e *Department of Medical Biochemistry, Faculty of Medicine, Afyonkarahisar Health Sciences University, 03200, Afyonkarahisar, Turkey.*; f *Departmentof Chemistry, Faculty of Arts and Sciences, Yildiz Technical University, 34220, Istanbul, Turkey. *; g *Departmentof Biology, Faculty of Science, Karamanoglu Mehmetbey University, 70100, Karaman, Turkey. *; h *Department of Histology and Embryology, Faculty of Medicine, Afyonkarahisar Health Sciences University, 03200, Afyonkarahisar, Turkey. *; i *Department of Biochemistry, Faculty of Medicine, Karabük University, 78020, Karabük, Turkey.*; j *Department of Medical Pharmacology, Faculty of Medicine, Afyonkarahisar Health Sciences University, 03200, Afyonkarahisar, Turkey.*

**Keywords:** Juglone, Resveratrol, Kefir, Ehrlich ascites, Bax, Bcl-2, p53, Caspase

## Abstract

We investigated the potential influence of kefir-induced juglone and resveratrol fractions (JRK) against Ehrlich Ascites Carcinoma (EAC) bearing BALB/c male mice. Kefir yeast was grown in the cell culture supplemented with juglone and resveratrol (1:2). After 48 h incubation, JRK solution was applied (0.1 mL/day i.p.) to the EAC-bearing mice throughout five days. Molecular regulatory mechanisms of apoptotic and anti-apoptotic pathway components were evaluated in the plasma of mice and isolated EAC cells with ELISA, qRT-PCR, and immunocytchemical experiments. EAC-induced upregulation in Bcl-2 and downregulation in Caspase-3 were normalized with JRK in the plasma of mice. Additionally, JRK upregulated the expression levels of apoptotic Bax, p53, Caspase-3,8,9, and APAF-1 proteins together with BAX, CASPASE-8, and CASPASE-9 genes in isolated EAC cells. These changes were also associated with decreased expression levels of anti-apoptotic Bcl-2 and Bcl-xl proteins. Immunocytochemical studies also confirmed the activation of apoptotic pathways and repression of anti-apoptotic proteins in EAC cells with JRK treatment. JRK activates apoptotic pathway and inhibits anti-apoptotic genes and proteins in Ehrlich ascites carcinoma- bearing BALB/c mice that could be beneficial in cancer treatment.

## Introduction

Cancer, a disease of genes directing the proliferation, differentiation, and the formation of cells, is the leading reason for death worldwide (1). Environmental and genetic factors determine the fate of cancer, which affects almost all body tissues, organs, and fluids (1, 2). Ehrlich Ascites Carcinoma (EAC) that could be developed from murine mammary spontaneously is modified to acidify form experimentally. This model can be kept going in outbred animals with serial intraperitoneal (i/p) passages. With their aggressive tumorigenic ability, EAC cells can rapidly proliferate and metastasize from peritoneal acid fluids to almost any body organ. Besides, they can also modulate some endogenous biochemical parameters in different body fluids, including serum and tissues (3, 4). Rodents’ peritoneal cavity might be used to passage and maintain the EAC cells, and the EAC model is recently used for tumor biology studies (5). The ability to monitor ascites carcinoma cells after injection to the animals, free neoplastic cell growing nature in abdominal fluids, and requirement of any mechanical stress for the disintegration of the cells make them as perfect cancer model for mechanistic cancer studies.

Recently, numerous bioactive compounds extracted from medicinal plants have been tested for their anti-cancer activity (6, 7). Several studies have reported the potential therapeutic effect of resveratrol (7, 8) and juglone (10-12) towards several cancer diseases. Pharmacological studies on resveratrol have reported its anti-oxidant, anti-inflammatory, anti-cancer, anti-aging, anti-obesity, anti-diabetic, cardio protective, neuroprotective, and anti-microbial properties (13). It also enhances the death of tumor cells by inhibiting Bcl-2 and upregulating Caspase-3 expression (14, 15). Furthermore, resveratrol could increase p53 and p21 by providing a balance between oxidative/anti-oxidative status (16, 17) and potentially causes cell death by inducing apoptosis. An irreversible inhibitor of the Pin1 (peptidyl-prolylcis/trans isomerase) enzyme; juglone is a plant-originated naphthoquinone which exhibits intense anticancer activity against numerous models *in-vitro* (18-21). Inhibition of Pin1 triggers mitogen-activated protein kinases that could contribute the cell survival (22, 23). Moreover, it is implicated in extensive diversity of clinical circumstances including immune response (24), allergy (25), cancer (26, 27), hyperparathyroidism (28), rheumatoid arthritis (29), and cardiac fibrosis (30-32). Recent studies revealed that juglone also induces apoptosis through the pathways dependent on mitochondrial pathways by the induction of the Bax/Bcl-2 ratio (33). Likewise, juglone increased Ca^2+^ concentration and Caspase-3 activity on human breast cancer cells (34). Its anti-tumor activity is usually linked to the damages on DNA due to alkylating species or oxygen radicals (35). Potentiation of tumor necrosis factor-α (TNF-α) related apoptosis in human melanoma cells via upregulating Caspase-3, p38, and p53 were also reported (36). Although juglone and/or resveratrol were shown to have anti-cancer effects in many *in-vitro* studies, promising results either alone or in combination in clinical studies have not been achieved yet.

Probiotics are currently defined as nonpathogenic microorganisms that improve human health due to their anti-inflammatory and immunoregulatory properties (37). Even though their action mechanism is not well-established, it is thought to be due to their metabolic products (38). A natural complex fermented milk product; kefir contains probiotic bacteria, primarily composed of Lactobacillus, Leuconostoc, Kluyveromyces, Pichia, and Saccharomycesspecies. It has anti-inflammatory, anti-allergenic, anti-oxidative, anti-tumor, cholesterol-lowering, constipation-lessening, and anti-microbial properties (39). Kefir also increases the anti-oxidant potential and regulates several biological processes such as apoptosis and proliferation (40, 41), and its apoptotic effects are attributed to the stimulation of Caspase-3 and Bax, repression of Bcl-2, and membrane potential reduction of mitochondria (42). Therefore, juglone, resveratrol, and kefir could have promising therapeutic potential against cancer treatments individually. However, in the current literature, there is no study showing the bioactive efficacy of combined juglone, resveratrol, and kefir solutions on cancer cell growth. Additionally, any study demonstrated the anti-cancer effects of secondary metabolites of kefir. In this study, we hypothesized that secondary metabolites of kefir rather than the bacterial part could have therapeutically properties. We also proposed that supplementation of kefir secondary metabolites with juglone and resveratrol and their potent secondary by-products obtained during the fermentation process could have strong pharmaceutical potential for cancer cell destruction. Therefore, this study was conducted to demonstrate the potent *in-vivo* apoptotic and anti-apoptotic effects of kefir secondary metabolites enriched with resveratrol and juglone and their potent by-products on of Ehrlich Ascites Carcinoma cells and of EAC-bearing BALB/c mice.

## Experimental

*Drugs and Preparation of the Solution JRK*

Chemicals for the bioassays were obtained from Sigma Aldrich (St. Louis, Missouri, USA) at the highest purity available. Juglone (H4700316) and resveratrol (EPRS-Y0001194) were dissolved in 0.1% DMSO, and the prepared mix has a ratio of 1:2, respectively. Kefir yeast contains Lactobacillus helveticus, Lactobacillus parakefiri, Lactobacillus casei, Lactobacillus reuteri, Lactobacillus acidophilus, Lactobacillus bulgaricus, Lactobacillus fermentum, Leuconostocmesentereoides, Lactococcuslactis, Lactobacillus kefiranofacien, and Acetobacterpasteurianus, Streptococcus thermophilus, Bifidobacteriumbifidu, Kluyveromycesmarxianus, Saccharomyces cerevisiae, and Kluyveromyceslactis were obtained from Danem-Kefir (Isparta, TURKEY). The bacteria in the kefir yeast were incubated in 10 mL of broth (Difco^TM^*Lactobacilli* MRS Broth 288130) for one night at 35 ^o^C and grown at the 1.5x10^+9^ cell/mL. During the fermentation process, juglone and resveratrol (200 µM-400 µM, respectively) were prepared (ethanol, <1% final concentration) and added to the broth and exposed for 48 h. The doses of juglone and resveratrol were determined according to parallel trials; resveratrol (43-45) and juglone (43, 46, 47) doses were chosen from previous *in-vitro* studies. Then, the solution was filtered through a 0.45 µm membrane, and the bacterial part was removed, and the remaining JRK solution was stored at -85 ^o^C until use. The numbers of bacteria were determined with trypan blue exclusion technique with Breed method. 

*Animals and Tumor Model*

Animal protocols were permitted in advance by the Local Ethics Commission for the Animal Research Studies at the Afyon Kocatepe University (AKUHADYEK-49533702.08). Male BALB/c inbred mice (18–22 g) were obtained from KOBAY A.Ş. (Ankara, TURKEY). Animals were kept growing in temperature-controlled rooms (20-22 °C) with a 12-h light-dark cycle. They were nourished with a typical rodent diet composed of 23% protein, 62% starch, 4% fat, 7% cellulose, standard vitamins, and a mixture of salt (chow pellet) and drinking water ad libitum. The EAC cells were gained from Afyonkarahisar Health Sciences University, Faculty of Medicine, Department of Anatomy (Afyonkarahisar, TURKEY). They were perpetuated with intraperitoneal (i/p) transplantation of 1x10^+6^ viable tumor cells in mice with a 25G needle to be used in future experimental studies. After five days of observation, JRK solution (0.1 mL/day i/p) was applied to the EAC treated mice for five days. Following the treatments, body weights and waist circumference of the mice were measured, and then ascites were isolated by a pre-scarification injector. The EAC cells’ viability was assessed with trypan blue exclusion technique (48). 

*Experimental Study Design*

After acclimation for one-week, male BALB/c inbred mice were randomly separated into three groups with ten animals each. Study protocol was designed as follows; Group (1): Non-EAC-bearing mice and untreated control group (Control), Group (2): EAC-bearing mice; injected 1×10^+6^ viable tumor cells in 1 mL of saline (Sham), Group (3): Applied JRK solution (0.1 mL/day i/p) to EAC-bearing mice for five days (JRK). Cancer modelling lasted five days. After this period, 0.1 mL saline was injected into the control and sham groups while applying the JRK solution.

*Measurement of Apoptotic/Anti-apoptotic Biomarkers in the Plasma and EAC*

Cardiac blood and EAC samples of mice were centrifuged immediately at 10.000 g for 30 min, and the cell pellet and supernatants were collected. Total protein levels in both plasma and EAC were determined by the Brilliant Blue G stain method using Sigma commercial kits (Saint Louis, Missouri, USA) as indicated in the package insert. The apoptotic Bax, p53, Caspase-3, Caspase-8, Caspase-9, and anti-apoptotic APAF-1 Bcl-2, Bcl-xl protein levels were determined by commercial rat specific ELISA kits (Cloud-Clone Corp., Export Processing Zone, Wuhan, Hubei, PRC) according to the manufacturer’s instructions. ELISA microplate reader (ChemWell 2910, Awareness Technology, Inc. Martin Hwy, Palm City) was utilized for the spectrophotometric measurements.

*Determination of the Expressions Levels of Apoptotic and Anti-Apoptotic Genes*

After the animal treatments, RNA samples were isolated from the tissue and EAC cells with a commercial RNA isolation kit (Qiagen, Netherlands) along with the manufacturer procedure. Quality and quantity were assessed with spectrophotometry and electrophoresis. Thereafter, cDNA synthesis was conducted using a commercial first-strand cDNA synthesis kit (Thermo Scientific, USA). BAX, CASPASE-6, CASPASE-8, and CASPASE-9 genes expression levels were determined with quantitative real-time polymerase chain reaction (Roche, Switzerland) as we described in detail previously (49). Briefly, SYBR Green Master Mix (Roche FastStart Universal SYBR Green Master Mix), cDNA samples, and the primer pairs (Table 1) were mixed, and the qRT-PCR was conducted. Triplicate measurements were performed for each sample, and the melt analysis confirmed the specificity of PCR reactions. For the normalization of data, internal control *gapdh* was utilized, and the relative expression of genes was calculated by LightCycler 480 SW 1.5.1 software (Roche, USA). 

*Immunocytochemical Analysis*

The suspended EAC cells were first pelleted by centrifugation at low speed for 5 min, and then, they were re-suspended in 4% formaldehyde in PBS. After warming up for 10 min at room temperature, EAC cells were pelleted again by centrifugation for 5 min at room temperature and resuspended in 80% ethanol. Fixed cells were stored at +4 ^o^C, and then the smears were stained with immunocytochemical (ICC) staining. For ICC, immobilized cells were incubated with 0.2% Tween-20 solution for 20 min for permeability. Afterward, the slides were washed with PBS and incubated for 10 min in 3% hydrogen peroxide/methanol solution. After washing, they were incubated with Bax (sc-526, Santa Cruz Biotechnology, 1/50 dilution), Bcl-2 (sc-7382, Santa Cruz Biotechnology, 1/50 dilution), p53 (sc-6243, Santa Cruz Biotechnology, 1/50 dilution), Caspase-3 (ab-4051, Abcam, 1/50 dilution) primary antibodies for 90 min. Then, HRP-conjugated secondary antibody kit (Anti-polyvalent HRP, Labvision Corp, Fremont, CA) was used to probe primary antibodies. After coloring with the chromogen, counter-staining with Mayer Hematoxylin staining was performed, and the slides were inspected using a light microscope. Cytoplasmic immunoreactivity for Bax, Bcl-2, and Caspase-3 and nuclear and cytoplasmic immunoreactivity for p53 were considered as specific. Bax, Bcl-2, p53, and Caspase-3 expressions were calculated as the percentage of positive cells out of the total cell number at five different randomly chosen areas from each slide under 400x magnification and the data are presented as percent positive cells.

*Statistical Analysis*

Data are represented as mean ± standard error of the mean throughout the manuscript. Student’s t-test or one-way ANOVA followed by the *Bonferroni* post hoc test were utilized where appropriate to determine statistically significant differences. The level of significance was set as *P* values which are smaller than 0.05.

## Results

*The Effects of JRK Solution Body Weight, Waist Circumference and EAC Cell Viability*

As shown in Table 2, Sham mice gained significantly higher weight and waist circumference than the control group (*P *< 0.05), whereas the terminal body weight and waist circumference of JRK-treated mice showed a tendency towards a decrease compared to Sham group. 

**Figure 1:**
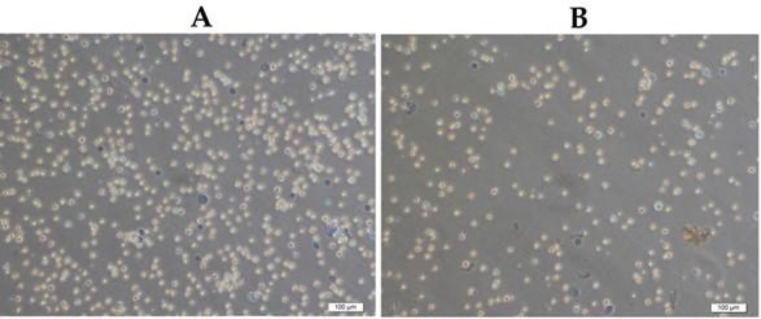
Ehrlich ascites carcinoma cells’distributions with the trypan blue exclusion method in Sham (A) and JRK (B) groups at the end of the experiment

Besides, the number of viable cells increased significantly (252.4 ± 18.4x10^+6 ^cell/mL) in EAC cells-injected (10^+6^) mice at the end of the 5^th^ day (Figure 1). Notably, JRK treatment reduced the number of viable EAC cells as compared to the Sham group (*P *< 0.05).

*The Effects of JRK Solution on Apoptotic/Anti-apoptotic Parameters in the Plasma and EAC Cells*

**Figure 2:**
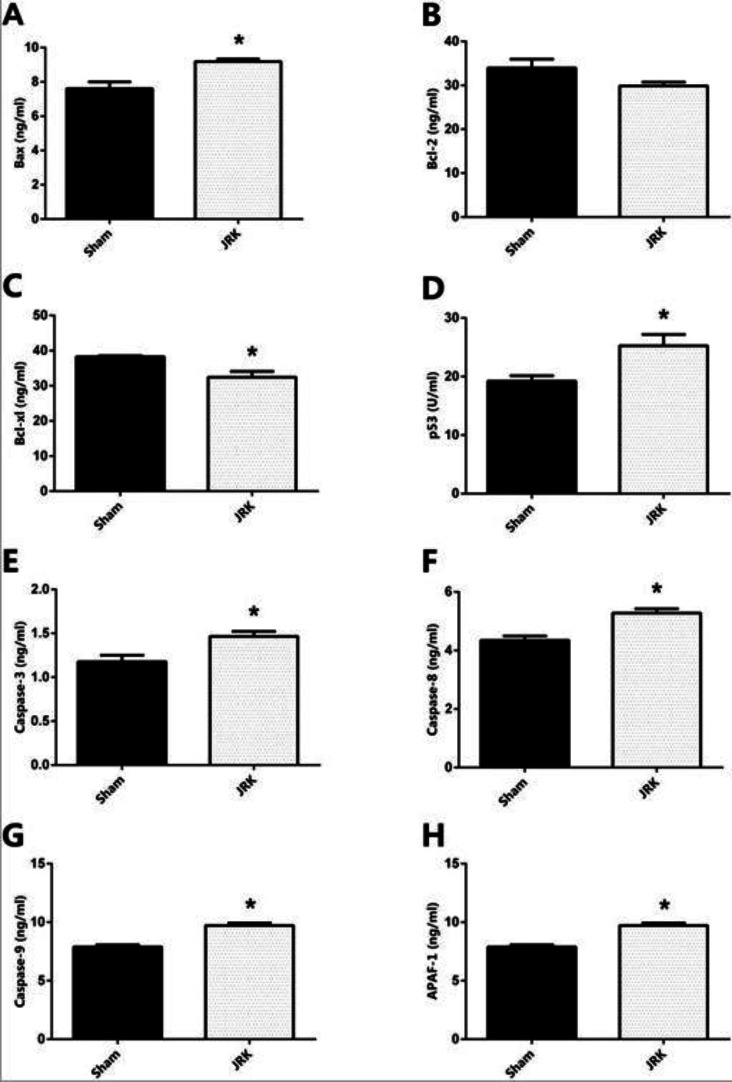
The protein expression levels of Bax (A) Bcl-2 (B) Bcl-xl (C) p53 (D) Caspase-3 (E) Caspase-8 (F) Caspase-9 (G) and APAF-1 (H) in the EAC cells of mice. Each bar represents the means of eight mice. Values are expressed as mean ± SEM ∗ significantly different (𝑃 < 0.05) compared to the sham group

**Figure 3:**
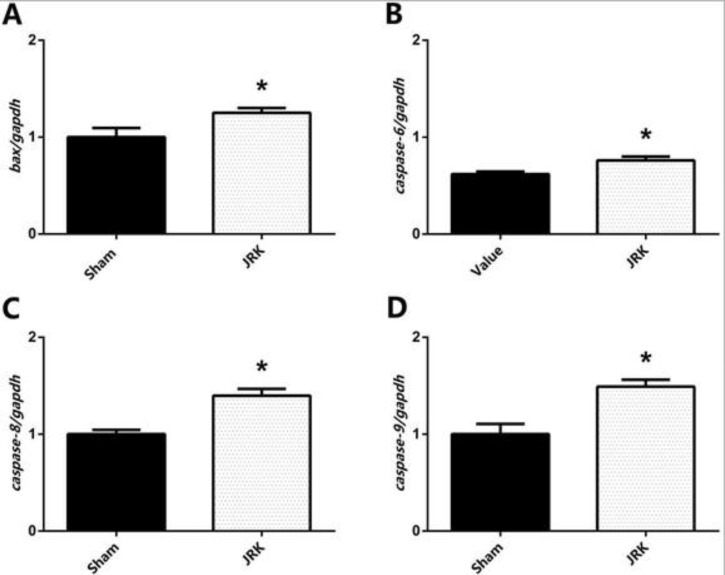
The mRNA expression levels of *BAX* (A), *CASPASE-6* (B), *CASPASE-8* (C), and *CASPASE-9* (D) in the EAC cell of mice. Each bar represents the means of eight rats. Data were normalized by *gapdh*. Values are expressed as mean ± SEM, ∗significantly different (𝑃<0.05) compared to the sham group

Apoptotic/anti-apoptotic biomarkers in the plasma of Sham and JRK supplemented group were evaluated with ELISA and qRT-PCR. Accordingly, the Bax, Bcl-xl, p53, Caspase-8, Caspase-9 and APAF-1 protein levels were all similar, while Bcl-2 was upregulated and Caspase-3 was downregulated in the plasma of Sham group. The changes in plasma Bcl-2 and Caspase-3 levels were normalized with JRK solution (data are not shown). Furthermore, there was a significant increase in Bax, p53, Caspase-3, 8, 9 and APAF-1 protein contents in the EAC-cell line of JRK-treated mice as compared to the sham group (Figure 2), while Bcl-xl levels were suppressed (Figure 2C) with JRK treatment. The mRNA expression levels of BAX, CASPASE-6, CASPASE-8, andCASPASE-9 were assessed in the EAC cell line, and the values are demonstrated in Figure 3. Accordingly, significantly higher levels of BAX (Figure 3A), CASPASE-6 (Figure 3B), CASPASE-8 (Figure 3C), and CASPASE-9 (Figure 3D) expressions were detected in JRK groups as compared to Sham. For technical reasons, it was not possible to detect CASPASE-3 gene expression in the EAC cells.

*The Influences of JRK Solution in the EAC cells: Immunocytochemical Evaluation*

**Figure 4:**
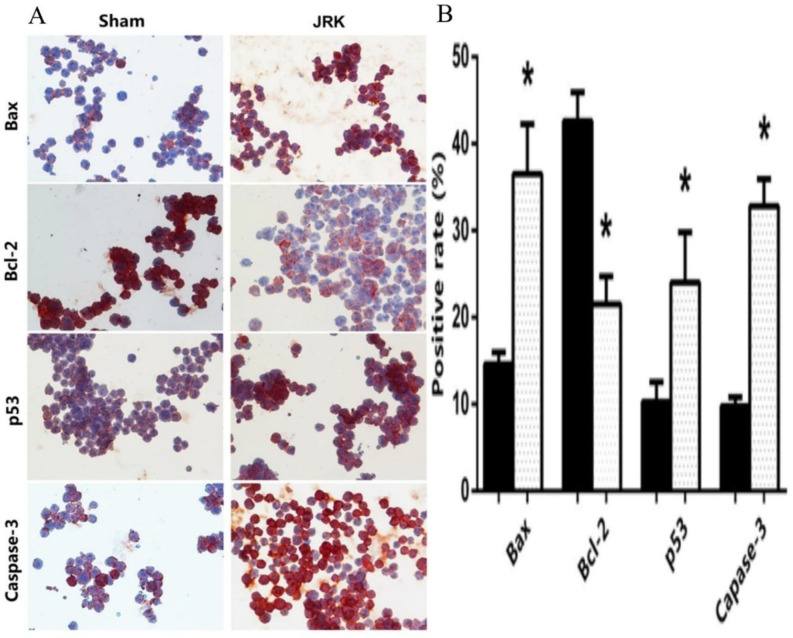
Immunocytochemical staining (x400 magnifications) (A) and quantitative analysis for the positive rate of cleaved Bax, Bcl-2, p53 and Caspase-3 expressions (B) in Sham (n = 6) and JRK (n = 7) rats. Values are expressed as mean ± SEM ∗significantly different (𝑃 < 0.05) compared to the sham group

Immunocytochemical examinations confirmed the diffuse and higher positive staining for Bax, p53, and Caspase-3 proteins in EAC cells of the JRK-treated group, but positive staining for Bcl-2 in the Sham mice (Figure 4A). As shown in Figure 4B, Bax (14.6 ± 1.4–36.5 ± 5.8), p53 (10.3 ± 2.3–26 ± 4.4), and Caspase-3 (9.75 ± 1–32.8 ± 3.1) positive rate of (%) EAC cells of JRK-treated mice were enhanced significantly compared to Sham group, whereas Bcl-2 (42.6 ± 3.3–21.5 ± 3.2) was suppressed.

**Table 1:** Primer sequences of bax, caspase-6, caspase-8, caspase-9, and internal standard *gapdh*used for the mRNA expression determination of qRT-PCR

**Gene**	**Reverse Primer**	**Forward Primer**	**Gene Bank Accession Number**	**Product Size (bp)**
*BAX*	GCTCAGCTTCTTGGTGGATG	CCTTTTTGCTACAGGGTTTCAT	NM-017059.2	110
*CASPASE- 6*	GACCGACTAAAACAGGCCC	AATTACTGTGCGCAAATGCC	XM-008761476.2	246
*CASPASE-8*	GCTGTAACCTGTCGCCG	ACCTCCGGTGTTTTATAGTTCC	NM-022277.1	179
*CASPASE-9*	ATGGTCTTTCTGCTCACCAC	GTCACGGCTTTGATGGAGAT	NM-031632.1	246
*GAPDH*	TCCTTGGAGGCCATGTGGGCCA	TGATGACATCAAGAAGGTGGT	NM-017008.4	240

**Table 2:** Initial and terminal body weight (BW), the number of viable cells, and the waist circumference (WC) of the experimental groups

**Groups**	**Initial BW (g)**	**Terminal BW (g)**	**WC (cm)**	**Cell Alive x10** ^+6^
Control	20.3 ± 0.9	21.8 ± 1.5	4 ± 0.1	-
Sham	20.9 ± 0.7	25.9 ± 1 #	11 ± 0.5 #	252.4±18.4
JRK	21.3 ± 0.4	25 ± 1.1	10.2 ± 0.4	119.1±7.4 *

Values (Control n: 6; Sham n: 8; JRK n: 8) are expressed as mean ± SEM, # significantly different (𝑃 < 0.05) compared to control group; ∗ significantly different (𝑃 < 0.05) compared to the sham group.

## Discussion

Cancer is a global problem of healthcare due to worldwide rising incidence and limited therapeutic options. Not only the ineffectiveness of treatments and side effects but also the complete remission in many cases prevents radical treatment of the disease (50). Therefore, it is essential to discover new therapeutic targets as well as new molecules with satisfying potential. Plant-derived compounds; juglone and resveratrol might be such potential therapeutic agents. Besides, dietary probiotics have also great potential for cancer prevention and treatment. Several studies have shown that juglone (10, 11, 18, 19, 33, 37, 51) resveratrol (8, 9, 12, 14-16) and kefir (41, 42) have positive effects on cancer treatment of colon, pancreas, lung, prostate, breast, and thyroid with human melanoma and myeloid leukaemia. Although the tremendous anti-cancer efficacy of the juglone through the Pin1 inhibition is well-known, its potent cytotoxic activity suppresses its selectivity (19). In this regard, taking juglone together with potent anti-oxidants would have a synergistic effect to reduce the cytotoxicity over healthy cells and to ensure appropriate pharmacotherapy (51). In this regard, we aimed to increase the selectivity of the juglone with a different strategy by investigating the effects of the end products of the kefir fermentation under juglone and resveratrol supplements.

Examination of the morphological alterations of the animals before decapitation indicated the body weight gain and waist circumference increment in the mice treated with EAC and JRK groups. This situation could be attributed to the increased intraabdominal fluid five days after the injection of EAC cells. Importantly, the number of viable EAC cells in the intraabdominal fluid from the mice in which the JRK solution is administered was reduced by more than 50% compared to the Sham group indicating the cytotoxic efficacy of JRK over EAC cells. These results are in parallel with the literature since juglone and/or resveratrol can induce apoptosis and repair DNA damage by affecting some endogenous factors. Exemplarily, juglone showed anti-cancer effect by inducing ROS/p38/p53 pathway in melanoma cells (36). 

Similarly, resveratrol and kefir induced expression of Bax, while inhibiting Bcl-2, and reinforcing the expression of p53 (52, 53). However, it was determined that consumption of kefir increased caspase-3 in myeloid leukemia cells (54). These parameters and mechanisms involved in cancer pathology can induce apoptosis. Our results also demonstrated the induction of apoptotic pathways and the suppression of anti-apoptotic markers in EAC cells of the mice treated with JRK-fractions. In EAC-cells, there was a general induction of apoptotic markers; Bax, p53, Caspase-3, 8, 9 and APAF-1 with JRK application. These inductions of apoptotic markers are also evidenced with the upregulated gene expression levels of BAX, CASPASE-6, CASPASE-8, andCASPASE-9. Besides, JRK downregulated the anti-apoptotic Bcl2-xl in EAC-cells of BALB/c in plasma of male mice. The present study also revealed proapoptotic Bax, p53, Caspase-8, Caspase-9, and APAF-1 and anti-apoptotic Bcl-xl levels do not alter in the serum of Sham group. However, upregulated Bcl-2 and suppressed Caspase-3 levels in the Sham group were normalized by JRK treatment.

In general respect, mitochondrial outer membrane permeabilization is an irreversible master regulator for the entry of cells towards the death which is strongly affected by synchronizing activities of Bax and Bcl-2 family proteins. Following the changes in membrane permeability, cytochrome C is released to the cytoplasm to activate particular proteases triggering apoptosis together with the APAF-1 association. In this process, while Bax potentially activates the apoptosis, Bcl-xl suppresses it by interacting with Bax (55, 56). Besides, cellular stress can also stimulate apoptosis via acetylation of p53 protein, which arrests the cell cycle under normal circumstances. Upon acetylation, p53 activates downstream proapoptotic target genes such as BAX. As a family of cysteine proteases and the master mediators of apoptotic cell death, Caspases are activated through cytochrome C release together with APAF-1 oligomerization (57, 58). Caspase-8 and Caspase-9 are initially activated with the cytochrome C and then lead to the proteolytic activation of other downstream proteases such as Caspase-3 and Caspase-6 which eventually shut down cellular functions by cleaving many proteins during the programmed cell death (59). The EAC modelling that we create shows first i/p distribution and systemic changes become apparent after a certain period; therewithal, we have set 10 days between the first injection and decapitation. There might have been any change in the subcomponents in the mechanism of apoptosis in the plasma. The modelling and the treatment that we applied cause changes in the essential apoptotic proteins; Bcl-2 and Caspase-3. This result means that systemic changes are in the initiation phase. In this context, JRK treatment decreased the anti-apoptotic Bcl-2 level and increased the apoptotic Caspase-3 level, which is a necessary consequence. In EAC cells, the proapoptotic factors; Bax, p53, Caspase-3, 8, 9 increased and anti-apoptotic Bcl-xl and Bcl-2 protein levels decreased in a synchronized way. These changes might induce the process leading to cell death. Although we used the JRK solution for the first time, other supporting literature information is available with that we can compare results. Firstly, resveratrol augmented the expression of apoptotic genes such as P53, BAX, CASPASE-3, and CASPASE-8 and promoted apoptosis with a pathway depending on mitochondria in breast cancer modelling (60). Similarly, resveratrol inhibited tumor angiogenesis as demonstrated by the reduction of microvessel density by vascular endothelial growth factor (VEGF) and VEGF receptor type-2 in the EAC-bearing mice (61). Moreover, juglone hindered EAC cells with reduced p-Akt levels (62). Another study presented the enrichment of BAX, CASPASE-3, CASPASE-8, and CASPASE-9 with juglone to inhibit the growth of breast cancer cells (63). Similarly, in our study, JRK solution amplified BAX, CASPASE-6, CASPASE-8, and CASPASE-9gene expressions as compared to Sham, which is in line with the change of their protein levels. Furthermore, the positive rate of the EAC cell line was examined by immunostaining of Bax, Bcl-2, p53, and Caspase-3. Staining of proapoptotic proteins Bax, p53, and Caspase-3 appear an increase following JRK-treatment, which is a probable compensatory mechanism against reduced Bcl-2. These results appear to be consistent with the protein and gene expression results of the measured parameters of the apoptotic mechanism.

## Conclusion

In conclusion, in this study, we highlighted the role, and the associated molecular mechanisms of JRK solution in cancer treatment, by mainly focusing on apoptotic mechanisms in EAC-bearing BALB/c mice. Even though this first stage study demonstrated that JRK is beneficial in cancer treatment and might be useful in clinical therapy, it necessitates additional clinical evidence. Further studies should be performed to determine the chemical composition of JRK solution, the secondary metabolites of kefir fermentation, juglone and/or resveratrol derivatives that might be formed through the fermentation process. To understand concrete molecular alterations of EAC and favourable effects of JRK, further studies should focuson the identification of JRK solution composition, long term, and dose-dependent response to JRK and determine survival rate and time. 
